# BODIPY Compounds Substituted on Boron

**DOI:** 10.3390/molecules29215157

**Published:** 2024-10-31

**Authors:** Marko Bogomolec, Mladena Glavaš, Irena Škorić

**Affiliations:** 1 Department of Organic Chemistry and Biochemistry, Ruđer Bošković Institute, Bijenička Cesta 54, 10 000 Zagreb, Croatia; marko.bogomolec@natur.cuni.cz (M.B.); mladena.glavas@irb.hr (M.G.); 2 Department of Organic Chemistry, Faculty of Chemical Engineering and Technology, University of Zagreb, Trg Marka Marulića 19, 10 000 Zagreb, Croatia

**Keywords:** BODIPY compounds, boron chemistry, fluorescent dyes, photocages

## Abstract

BODIPY compounds are important organic dyes with exceptional spectral and photophysical properties and numerous applications in different scientific fields. Their widespread applications have flourished due to their easy structural modifications, which enable the preparation of different molecular structures with tunable spectral and photophysical properties. To date, researchers have mostly devoted their efforts to modifying BODIPY *meso*-position or pyrrole rings, whereas the substitution of fluorine atoms remains largely unexplored. However, chemistry of the boron atom is possible, and it enables tuning of the photophysical properties of the dyes, without tackling their spectral properties. Furthermore, modifications of boron affect the solubility and aggregation propensity of the molecules. This review article highlights methods for the preparation of 4-substituted compounds and the most important reactions on the boron of the BODIPY dyes. They were divided into reactions promoted by Lewis acid (AlCl_3_ or BCl_3_), or bases such as alkoxides and organometallic reagents. By using these two methodologies, it is possible to cleave B–F bonds and substitute them with B–C, B–N, or B–O bonds from different nucleophiles. A special emphasis in this review is given to still underdeveloped photochemical reactions of the boron atom of BODIPY dyes. These reactions have the potential to be used in the development of a new line of BODIPY photo-cleavable protective groups (also known as photocages) with bio-medicinal and photo-pharmacological applications, such as drug delivery.

## 1. Introduction

**Figure 1:**
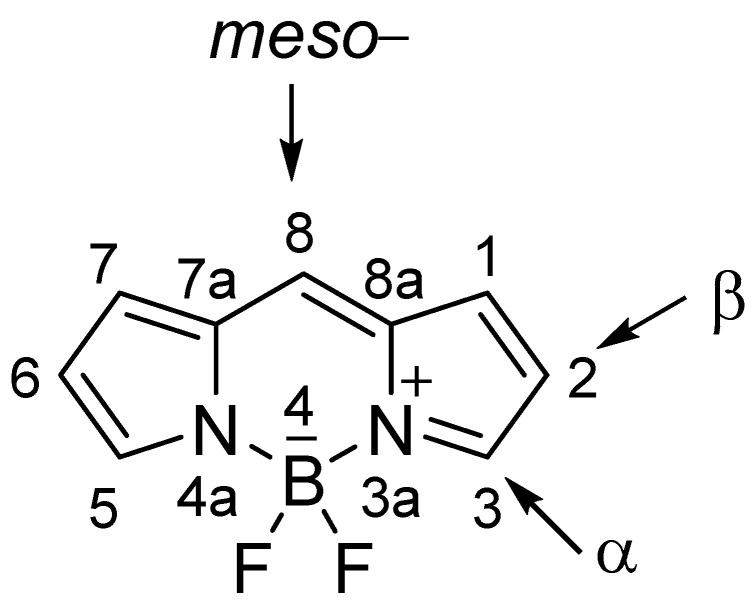
Structure of BODIPY with the numeration according to the IUPAC.

BODIPY is the commercial name for a class of heterocyclic fluorescent compounds with the IUPAC name 4,4-difluoro-4-bora-3a,4a-diaza-*s*-indacene (Figure 1) [1], that were discovered by A. Treibs and F.H. Kreuzer in 1968 [2]. To date, they have received a significant scientific interest owing to their exceptional photophysical properties and widespread use in material science [3], fluorescence sensing [4], molecular biology, and medicine, where they have been used as fluorescent markers [5,6,7] and phototherapeutics [8,9,10,11,12,13]. Their use in different applications has been primarily enabled by their easy structural modifications, which allow for tuning of spectral and photophysical properties [14,15]. Consequently, the chemistry of BODIPY compounds has been reviewed on several occasions [16,17,18,19,20], albeit the reviews on the reactions on boron in BODIPYs are scarce [21,22,23]. Noteworthy are some review articles that also discussed the 4-substituted BODIPY compounds [24,25,26,27,28,29,30,31]. However, to date no review article covers systematic description for the preparation of differently 4-substituted BODIPY compounds, together with their spectral and photophysical properties, which is the topic of the current mini-review.

BODIPY compounds generally show chemical and photochemical stability, good solubility in most organic solvents, a low tendency for self-aggregation in solution and stability in physiological conditions. They are characterized by intense and narrow absorption bands in the visible part of the electromagnetic spectrum that correspond to the 0-0 transition and the population of the S_1_ state, and a less pronounced shoulder attributed to the 0-1 vibrational transition [4,14,15,16,17,18,19,20]. The absorption maxima, *λ*_abs_, are most often in the range 470–530 nm and they weakly depend on the polarity of solvent [4,14,15,16,17,18,19,20]. The values of their molar absorption coefficients are high, in the range of 40,000–110,000 M^−1^ cm^−1^ [4,14,15,16,17,18,19,20]. Most of them have large values of fluorescence quantum yields, with singlet excited state lifetimes of 1–10 ns, and negligible radiationless deactivation and the population of the triplet excited states [32].

**Scheme 1:**
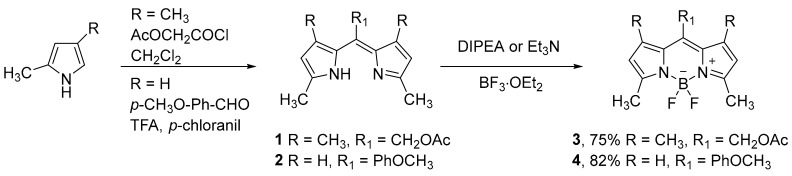
Synthesis of BODIPY from a pyrrole derivative and acyl chloride or aromatic aldehyde.

The synthesis of BODIPY derivatives is based on the preparation of dipyrromethenes, which are subsequently complexed with BF_3_ in the presence of a base [2,16,17,18,19,20]. By using substituted pyrrole and suitable acid chloride, in the first steps of the synthesis, alkylated dipyrromethene **1** is prepared, which is converted into BODIPY compound **3** (Scheme 1) [33].

The influence of the alkyl groups on spectroscopic and photophysical properties is negligible, but alkyl substituents are useful for further functionalization. The other often used procedure for the preparation of differently substituted dipyrromethenes is based on the MacDonald method, from pyrrole and pyrrole carbaldehyde derivatives, usually in the presence of acids such as HBr [34,35]. Derivatives with the desired aryl groups at the position 8 are easily prepared by choosing an appropriate aromatic aldehyde in the synthesis of dipyrromethane **2** [36], which are subsequently oxidized, typically with DDQ or *p*-chloranil (Scheme 1) [16,17,18,19,20,37]. Substituents in the *meso*-position do not have a significant effect on the wavelengths of absorption and emission maxima, but they can modify the fluorescence quantum yields [4,16,17,18,19,20]. If the aromatic ring contains electron-donating groups such as tertiary amines, or electron-withdrawing groups such as nitro- or cyano-groups, additional photophysical processes in the excited state, such as photoinduced electron transfer (PET) or charge transfer (CT), affect the fluorescence [4]. Furthermore, phenyl groups in the *meso*-position greatly reduce fluorescence quantum yields if their rotation is not sterically hindered [38,39,40], which was applied in the development of molecular rotors—fluorescent probes for microviscosity and temperature [41,42].

The great advantage of BODIPY compounds compared to other fluorophores is the large number of possible synthetic modifications of the chromophore, which lead to changes in spectral and photopysical properties [14,15]. It is possible to introduce the desired substituents and carry out reactions on the entire chromophore—pyrrolic carbon atoms, *meso*-carbon, and the boron atom.

A frequent modification of BODIPY compounds is the introduction of halogen atoms as reactive groups. Halogenated derivatives are suitable because they enable the introduction of target functional groups in the late steps of the synthesis [43]. A large number of halogenation reactions have been described: bromination with elemental bromine [44], *N*-bromosuccinimide [45], and CuBr_2_ [46], chlorination with *N*-chlorosuccinimide [47], and CuCl_2_·2H_2_O [48], iodination with ICl [49] and I_2_/HIO_3_ [50], or *N*-iodosuccinimide. Most importantly, the use of copper halogenides gives rise to selective halogenation. Thus, CuBr_2_ yields the brominated positions 2 and 6, whereas by use of CuCl_2_, chlorines enter the positions 3 and 5. The difference in regioselectivity is due to different reaction mechanism in the case of CuCl_2_, where single-electron transfer takes place.

**Scheme 2:**
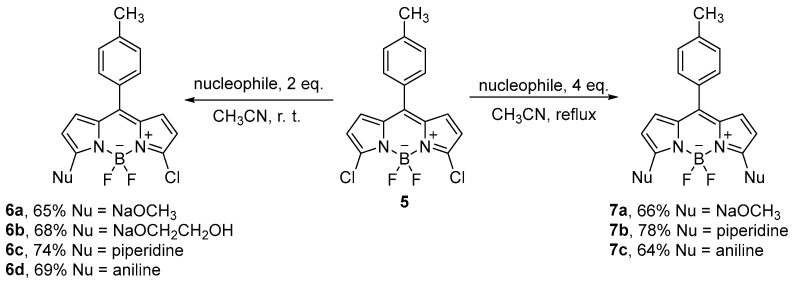
Nucleophilic aromatic substitution on BODIPY **5**.

The presence of bromine or iodine atoms in the BODIPY structures increases the rate of intersystem crossing due to greater spin-orbital coupling owing to the heavy atom effect, and consequently, there is a decrease in fluorescence quantum yields [51]. The population of triplet excited state enables the generation of singlet oxygen, which is the main condition for the use of BODIPY compounds in photodynamic therapy where the iodo-derivatives show the greatest potential [8,9,10,11,12,13]. Moreover, halogenated BODIPY derivatives can undergo reactions characteristic of halogenated aromatic heterocycles, such as nucleophilic aromatic substitution (Scheme 2). Substitution reactions can be carried out with carbon, oxygen, and nitrogen nucleophiles [52].

**Scheme 3:**
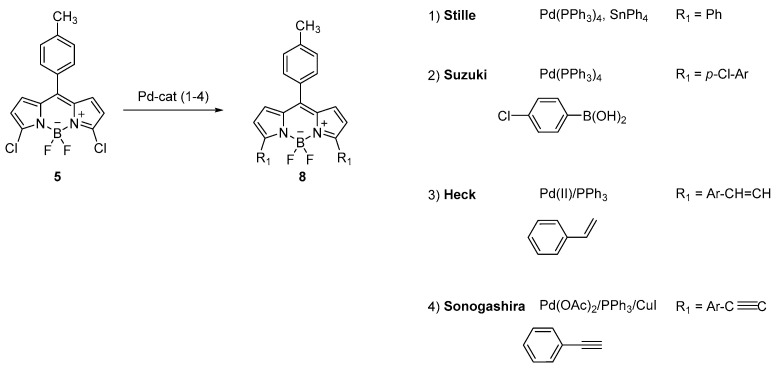
Pd-catalyzed cross-coupling reactions on chlorinated BODIPY **5**.

Halogenated BODIPY compounds, including chloro-derivatives, undergo palladium-catalyzed cross-coupling reactions, such as Suzuki, Stille, Heck, and Sonogashira reactions (Scheme 3) [43,53,54], which have been extensively used to enlarge chromophoric systems and shift the absorption bathochromically to red and infrared regions [14,15,16,17,18,19,20]. The smallest bathochromic shift in the absorption spectrum is caused by a phenyl substituent, and the largest by a styryl substituent. Note that palladium-catalyzed reactions also proceed with molecules bearing halogens at other positions than 3 and 5. Moreover, it should be emphasized that non-halogenated compounds can also undergo palladium-catalyzed cross-coupling reactions via C-H- insertions [55].

Furthermore, alkenyl derivatives are more often prepared from BODIPY compounds bearing methyl substituents at the position 3 and 5 by Knoevenagel reactions with aromatic aldehydes [50,56,57,58,59]. It is interesting to note that BODIPY compounds with alkenyl substituents generally have high fluorescence quantum yields and long singlet excited state lifetimes, even though it is known that alkenes very effectively deactivate by photochemical *E*-*Z* isomerization [60]. Namely, the excitation of alkenyl BODIPY derivatives to S_1_ does not lead to a decrease in the electron density between the C-atom of the double bond, which would result in the torsional relaxation on the S_1_ surface [61].

## 2. Direct Synthesis of 4-Substituted BODIPYs from Dipyrromethene Derivatives

As BODIPY is the name for derivatives of 4,4-difluoro-4-bora-3a,4a-diaza-*s*-indacene, it is understood that two fluorine atoms are attached to the boron atom in the structures. However, reactions have been developed by which it is possible to derivatize the 4- position, that is, the substitution reactions of fluorine on the boron atom have also been developed [14,15,21,22,23,24,25,26,27,28,29,30,31].

**Scheme 4:**
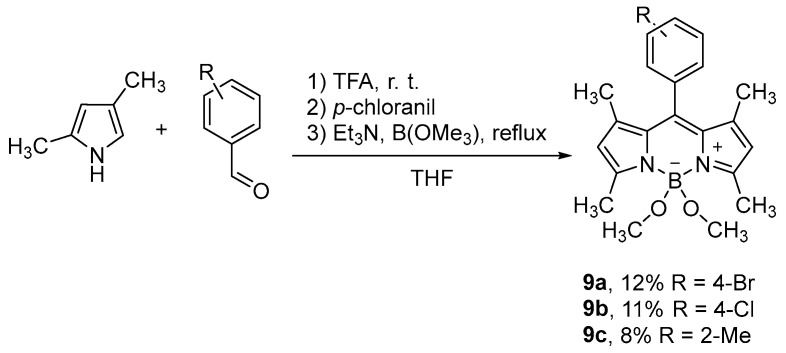
Synthesis of dimethoxy BODIPY compounds **9**.

BODIPY compounds substituted on the boron can be prepared already in the first steps of the synthesis. The BODIPY prepared from 2,4-dimethylpyrrole and aromatic aldehydes, with subsequent oxidation with *p*-chloranil to dipyrromethene and the treatment with triethylamine and trimethylborate as Lewis acid (instead of BF_3_), gave compound **9** in low yields (Scheme 4) [62].

**Scheme 5:**
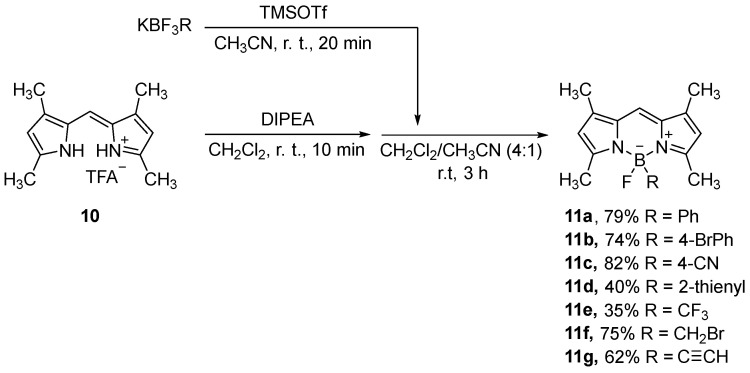
Synthesis of 4-substituted BODIPY derivatives **11**.

Higher yields on the 4-substituted (B-substituted) products, and wider scope of the 4-substituted substrates can be obtained in the reactions of dipyrromethenes with potassium salts of 4-substituted trifluoroborates (Scheme 5) [63]. The reaction afforded 4-monoalkylated or monoarylated compounds **11** in moderate to good yields, as well as perfluoroalkyl derivatives, which were used in the live cell imaging [64,65] or inhibition of tau amyloid formation [63].

**Scheme 6:**
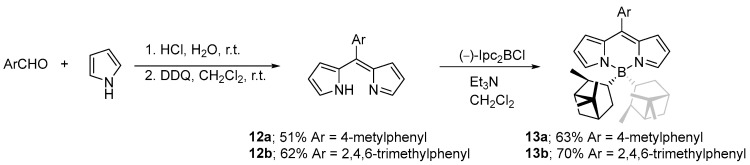
Synthesis of 4,4-diisopinocampheylBODIPYs **13a** and **13b**.

*C*-BODIPY compounds **13**, which are substituted on boron with isopinocampheyl groups, can be prepared from dipyrromethene **12** using chloro(diisopinocampheyl)borane (Ipc_2_BCl) under basic conditions (Scheme 6) [66]. Compound **13a** was the first published BODIPY dye with stereogenic carbon centers directly linked to the boron atom. Compound **13b** was prepared analogously **13a**, but with *ortho*-methyl groups at the *meso*-phenyl substituent, to avoid the free rotation around the BODIPY-phenyl bond. Compound **13** was used as circularly polarized luminescence (CPL)-active boron chelate [66].

## 3. Reactivity of BODIPY Compounds on Boron Under Thermal Conditions

In addition to the preparation of BODIPY compounds with substituents at the boron by use of different boron-complexing agents (previous section), the transformation of BODIPY position 4 is also possible by substitution of the fluorine. The reactions can be generally divided into those carried under basic conditions and the Lewis-acid promoted.

**Scheme 7:**
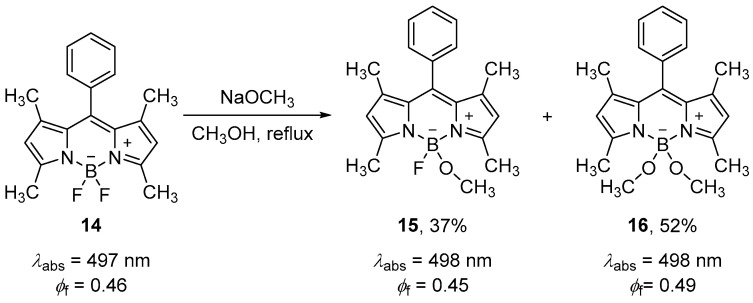
The substitution of fluorine in BODIPY by alkoxide, maxima in the absorption spectra and quantum yields of fluorescence.

Oxygen nucleophiles can substitute fluorine at the boron atom under basic conditions, often at high temperatures. Thus, the reaction of compound **14** with sodium methoxide in methanol gave a mixture of compounds **15** and **16** (Scheme 7) [67]. The BODIPY compounds with alkoxy substituents at the boron have similar spectroscopic properties as the unsubstituted BF_2_ compound, but their oxidation potentials differ. By replacing the more electronegative fluorine with a methoxide group, the inductive effect of fluorine is weakened, and the oxidation potentials have lower values by about 0.1 V per OCH_3_ group. Therefore, the substitutions on boron have potential to tune properties of molecules for PET.

Fluorine substitution reactions can also be conducted with sterically demanding nucleophiles, e.g., potassium *tert*-butoxide, but they are often limited to monosubstitution and have low reaction yields [68]. The use of a large excess of nucleophiles in the reaction does not give better yields on the substitution products, but results in the elimination of BF_2_ and release of the free dipyrromethenes.

### 3.1. Lewis Acid Promoted Formation of the B–O and B–N Bonds

**Scheme 8:**
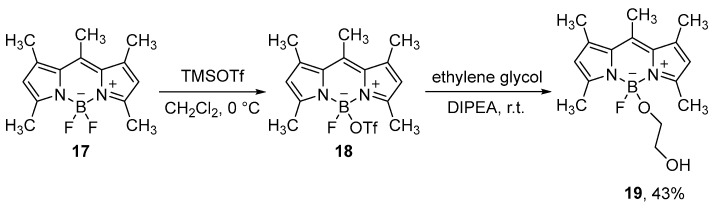
Activation of the B–F bond in compound **17** with TMSOTf and preparation of alkoxy-BODIPY compound **19**.

The use of Lewis acids, which interact with the boron atom or fluorines in BODIPY compounds, allows for the substitution of fluorine with different groups. Thus, R. Mazitschek et al. developed a two-step, one-pot method for the synthesis of a series of 4-alkoxy-BODIPY derivatives. Selective monosubstitution is carried out on BODIPY **17** by the use of trimethylsilyl-trifluoromethanesulfonate (TMSOTf), which binds to boron and forms an activated intermediate **18**, which reacts with alcohols (Scheme 8). The intermediate product is not stable under the reaction conditions and dissociates slowly, forming free dipyrromethene, so an alcohol must be added to the reaction mixture at the appropriate time [69].

**Scheme 9:**
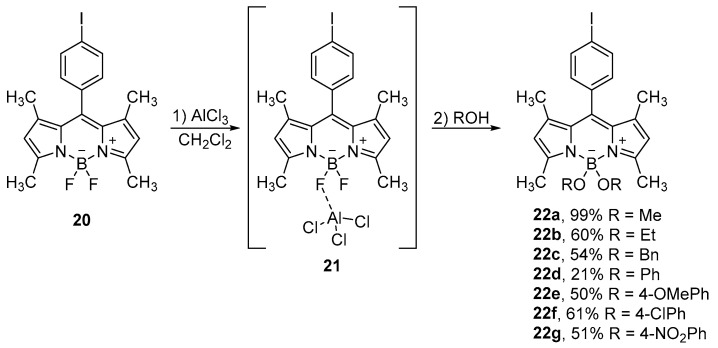
The substitution of fluorines in BODIPY **20** with alkoxides in the presence of AlCl_3_ as a Lewis acid, which binds to the fluorine and forms an activated complex **21**.

**Figure 2:**
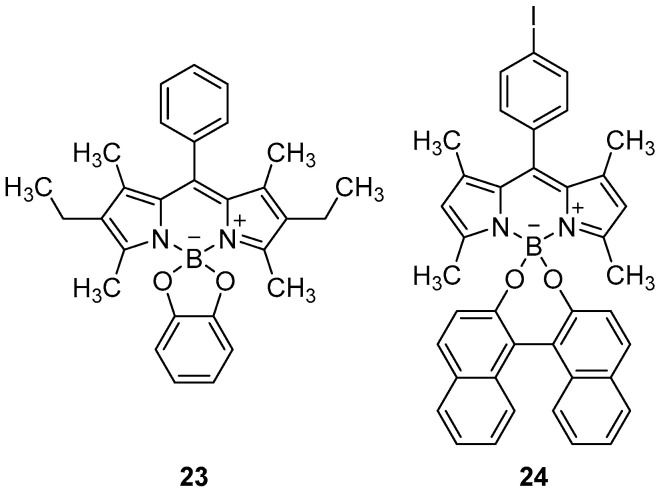
Cyclic nonfluorescent BODIPY compounds obtained by AlCl_3_-catalyzed reactions with dihydroxy aromatic derivatives.

A convenient substitution method for the activation of the B–F bonds is based on the use of AlCl_3_, which proved to be compatible with a large number of functional groups. Such a synthetic protocol was used for the preparation of 4,4-dialkoxy and 4,4-diaryloxy-BODIPY compounds **22** in the reactions with alcohols and phenolic derivatives (Scheme 9). The reaction conditions were tolerant for the aldehydes, esters, or amino groups [70]. If dialcohols or dihydroxybenzene derivatives such as catechol are used for the substitution, both fluorine atoms can be substituted yielding cyclic derivatives (Figure 2) [71].

Furthermore, the authors reported that the fluorescence of the catechol derivative **23** (Figure 2) was quenched by very efficient PET from the catechol unit to the BODIPY chromophore, which can be restored by substitution of the catechol by methoxides.

**Figure 3:**
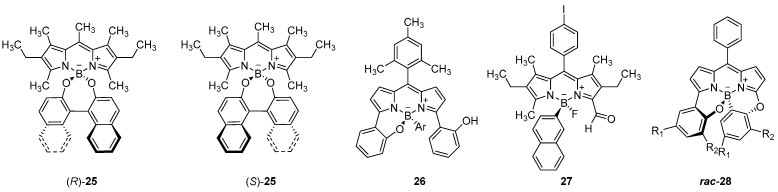
4-substituted chiral BODIPY compounds.

AlCl_3_-promoted intramolecular cyclization reactions with phenol derivatives were used in the synthesis of a number of 4,4-disubstituted compounds with axial chirality [24,25,26,27,28,29,30,31]. They were prepared as fluorophores exhibiting circularly polarized luminescence (CPL) [72]. For example, enantiomers **25** in Figure 3 show perfect mirror image symmetry in the CPL spectra, although the intensity is not high [73,74]. Furthermore, intramolecular cyclization of phenolic derivatives afforded compounds **26** and **27** shown in Figure 3 with the chiral B-atom [75,76]. Chiral structures with the asymmetric boron atom have recently received significant attention [77]. Moreover, M. J. Hall et al. prepared a series of helically chiral molecules such as **28** by Pd-catalyzed arylations of dypyrromethene with hydroxyphenyl boronic acids that exhibited CPL [78]. Chiral molecules exhibiting aggregation induced circularly polarized emission have also been reported [79].

**Scheme 10:**
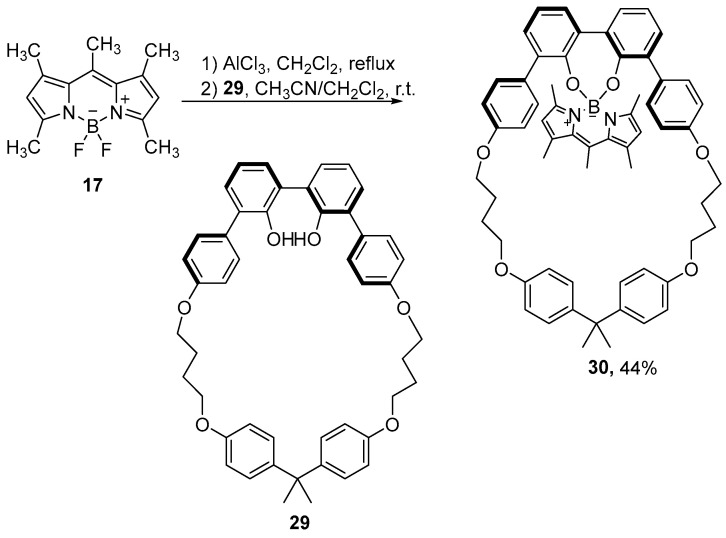
Synthesis of threaded BODIPY **30** by 4-substitution.

The AlCl_3_-promoted reaction of BODIPY with a cyclic bisphenol was used to prepare threaded BODIPY **30** (Scheme 10) [80]. Compound **30** showed excellent photophysical properties with a high fluorescence quantum yield. Moreover, incorporation of the BODIPY in the macrocycle hampers its oxidative oligomerisation. Furthermore, F. D’Souza et al. used AlCl_3_ to promote cyclization with catechol to prepare BODIPY-fullerene dyads capable of harvesting light energy from the near-IR region that undergo ultrafast charge separation [81].

**Scheme 11:**
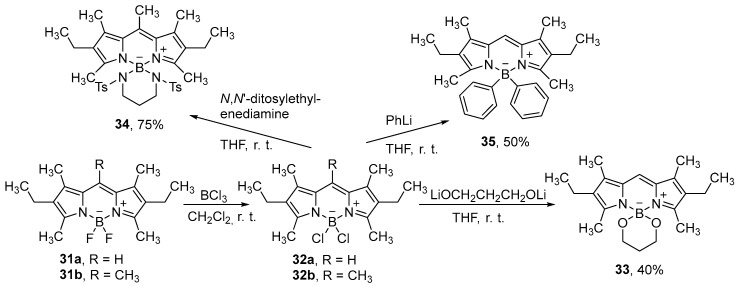
Synthesis of 4,4-dichloro BODIPY compounds **32** and subsequent substitutions with nucleophiles.

A. Thompson et al. described the reactivity of BODIPY compounds with BCl_3_ [82,83,84]. The addition of boron trichloride to the solutions of BODIPY **31** quantitatively results in the formation of 4,4-dichloro-BODIPY compound **32** (Scheme 11). The chlorinated BODIPY derivatives are very reactive compounds, but they can be isolated under inert conditions. Fluorescence quantum yields of the chlorinated derivatives are lower than fluorinated analogs, plausibly due to the heavy atom effect. The lower strength of the B–Cl bonds makes them more susceptible to nucleophilic attacks and enables fast and efficient substitutions with C-, N- or O-nucleophiles (Scheme 11) [82,83,84,85].

**Scheme 12:**
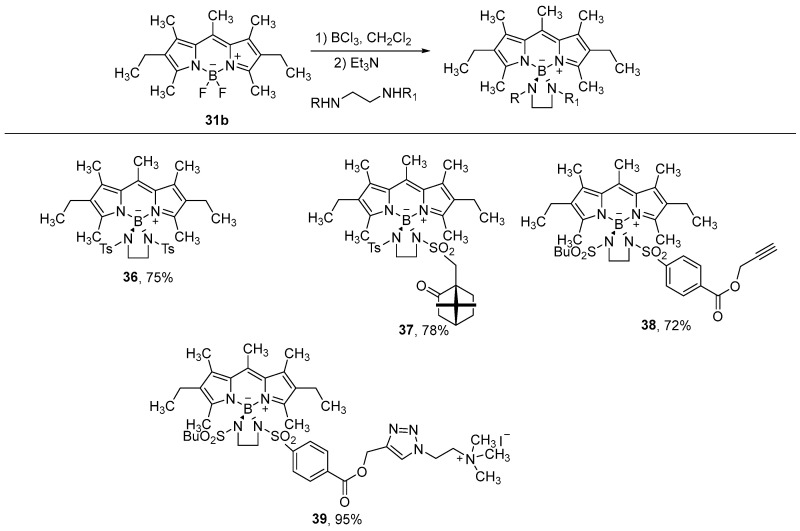
Synthesis of unsymmetrical BODIPY derivatives **36**–**39**.

The transformation of the activated BCl_2_ molecule with disulfonamides was also used for the preparation of a series of unsymmetrical BODIPY derivatives **36**–**39** (Scheme 12) [86]. The molecules were prepared with the aim to develop specific bioprobes for fluorescent imaging, chromophores that exhibit circularly polarized luminescence in the visible spectrum or to act as efficient multichromophoric arrays for light harvesting by excitation energy transfer. Compound **36** was designed for acting in lasing, **37** as an efficient chiroptical BODIPY based on sustainable camphor, **38** as a key intermediate to BODIPY dyes for cell-imaging, and **39** as potential photodynamic therapy (PDT) agent. Interestingly, all synthesized molecules have similar photophysical properties with quantum yields of fluorescence in the range *Φ*_F_ = 0.8–0.9 regardless of the substituents at the boron [86].

**Figure 4:**
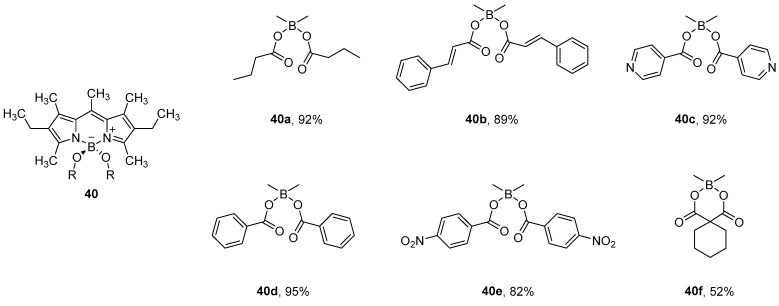
Different B-acyl derivatives **40** prepared by the BCl_3_ activation protocol [88].

The activation of BODIPY compounds by BCl_3_, and subsequent quenching of the reactive BCl_2_ molecule with different acyl derivatives was used by S. de la Moya et al. [87]. They prepared a series of BODIPY compounds **40** (Figure 4) for applications as laser dyes.

**Scheme 13:**
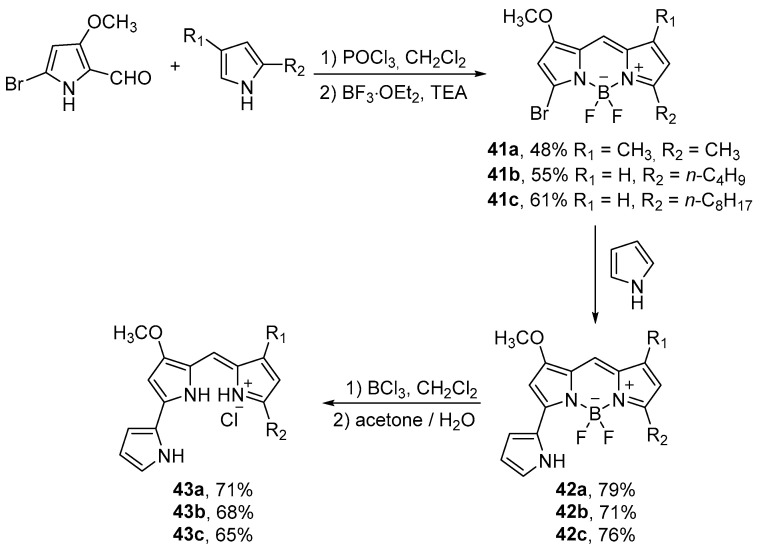
Synthesis of prodigiosene derivatives **43**, where the decomplexation of BODIPY by use of BCl_3_ was used as the key step.

The use of BCl_3_ can also lead to the elimination of BF_2_ from BODIPY and formation of the free base dipyrromethene [89]. Such a strategy was used to prepare natural compounds prodigiosene derivatives **43** (Scheme 13) [88].

### 3.2. Substitutions at the Boron by Use of Trimethylsilyl Reagents

**Scheme 14:**
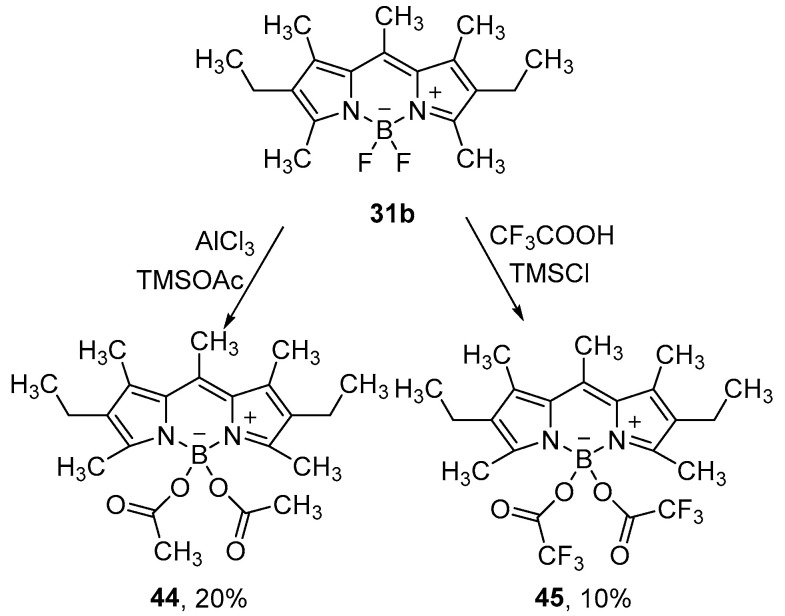
Substitution of fluorines in BODIPY **31b** with carboxylic groups.

In addition to alcohols, carboxyl groups can also be attached to the boron atom. Substitutions with carboxyl groups are carried out by using trimethylsilyl carboxylates generated in situ from carboxylic acids and trimethylsilyl chloride [90]. Thus, 4,4-diacetoxy-BODIPY compounds were prepared in good yields in the reactions with trimethylsilyl acetate in the presence of AlCl_3_ as a Lewis acid (Scheme 14) [90]. The acyloxy groups are located perpendicular to the plane defined by the planar BODIPY structure. Therefore, this substitution does not affect the absorption and emission maxima, but it significantly improves the solubility in polar solvents [91].

**Scheme 15:**
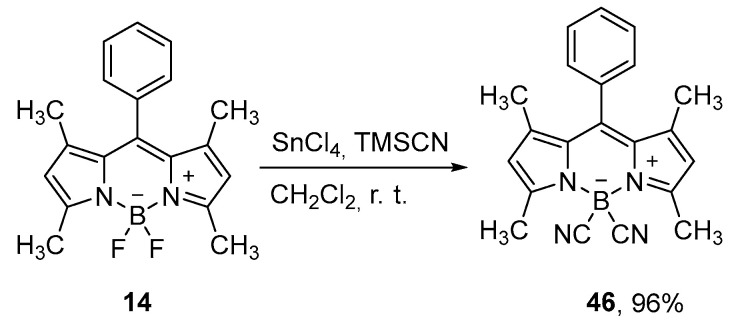
Substitution of fluorine in BODIPY **14** and preparation of a cyano derivative **46**.

By use of trimethylsilyl reagents it is also possible to substitute fluorines by cyano groups. Thus, BODIPY **14** in the presence of SnCl_4_ and trimethylsilyl cyanide (TMSCN) gives **46** in excellent yield (Scheme 15) [92]. The same authors also studied acid stability of differently 4-substituted BODIPY compounds. They found out that the cyano substituted compound such as **46** is more resilient to TFA, compared to 4,4-dialkyl- or 4,4-dialkoxy-substituted BODPY compounds, which they associated with higher aromaticity of the cyano substituted derivative [78].

**Scheme 16:**
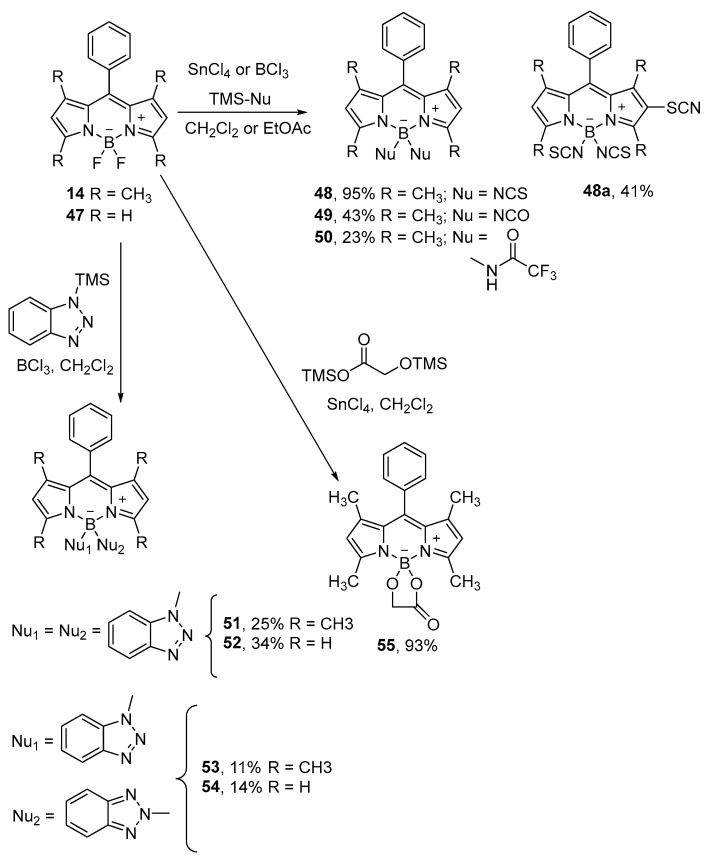
The use of TMS reagents to substitute fluorine in BODIPY **14** and **47**.

Similarly to the cyanation, trimethylsilyl (TMS) reagents were used to substitute the fluorines by SCN, benzotriazoles or cyclic esters (Scheme 16) [93]. Different substituents did not affect the spectral properties of the compounds but changed fluorescence quantum yields.

**Scheme 17:**
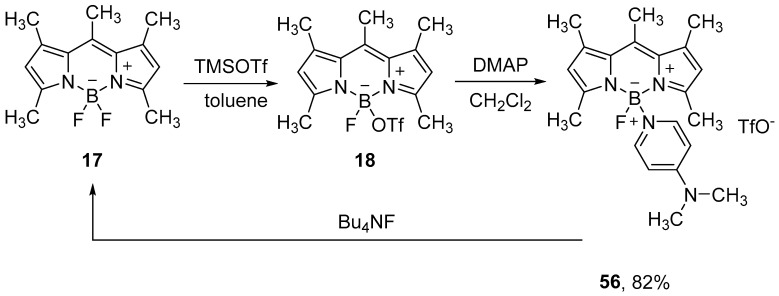
Synthesis of fluoride PET sensor **56** and its reaction with F^−^, which increases fluorescence.

Nonfluorescent alkoxy BODIPY derivatives, such as **56**, can be used as a PET sensor for fluoride anions. In the presence of F^−^, the parent BODIPY compound **17** is restored, which results in a significant increase in fluorescence (Scheme 17) [94].

### 3.3. Organometallic Alkylation and Arylation

**Scheme 18:**
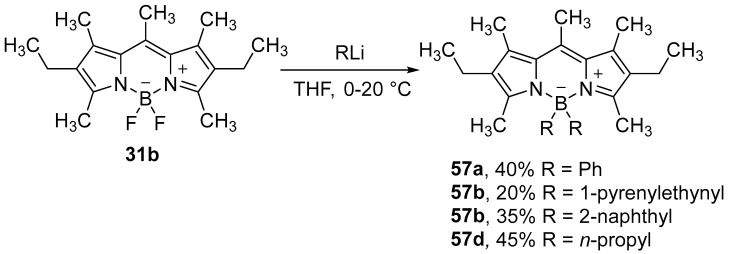
Substitution of fluorine in **31b** by organolithium reagents.

Strong carbon nucleophiles, such as organolithium compounds, attack the boron center of BODIPY compounds and substitute the fluorine atoms. Such a methodology was used for the synthesis of 4-alkyl [95], 4-aryl [89,96], and 4-alkynyl [97] BODIPY derivatives (Scheme 18). Organolithium reagents enable efficient substitution even under mild reaction conditions, but due to vigorous reactivity, monosubstituted derivatives cannot be formed [89].

The absorption spectrum of the disubstituted compound **57a** is similar to the overlapped absorption spectra of the BODIPY chromophore in **31b** and the pyrenylalkyne, indicating no electron delocalization over the boron center. Furthermore, the photoexcitation of the pyrenylalkynyl groups gives rise to the emission from the BODIPY fluorophore. A good overlap of the S_0_→S_2_ bands of the BODIPY absorption and the pyrene emission enables efficient Förster resonance energy transfer (FRET) from the pyrene to the BODIPY [97].

**Scheme 19:**
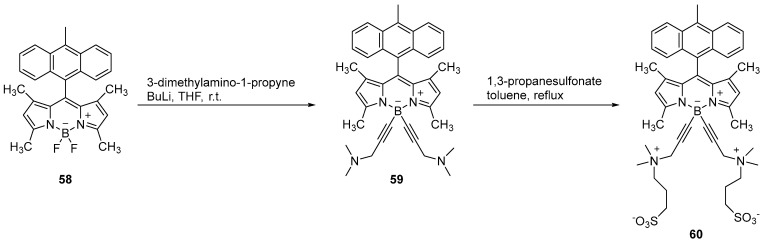
The use of organometallic reagents in the preparation of H_2_O-soluble BODIPY **60**.

4,4-Disubstituted BODIPY **59** was prepared in the reaction of acetylide, made in situ from alkyne and BuLi (Scheme 19). Compound **5** was further transformed into a H_2_O-soluble BODIPY **60**, which was used for the production and visualization of singlet oxygen in breast cancer cells [98].

**Figure 5:**
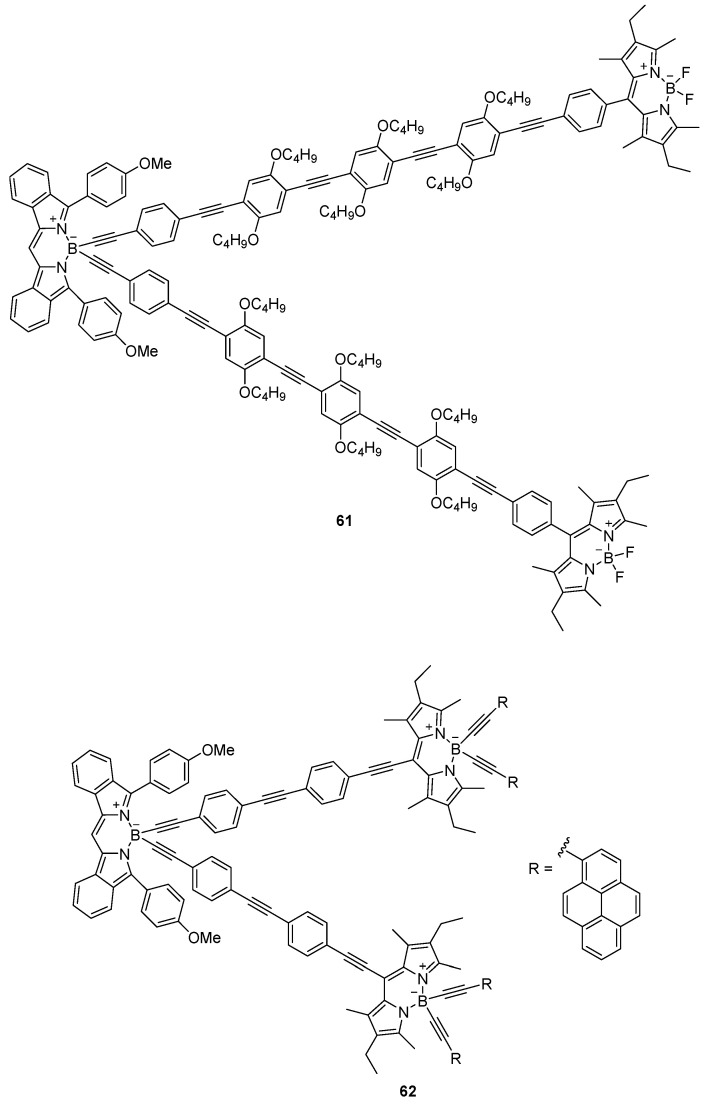
4-substituted compounds **61** and **62**, which were used as four-color donor-acceptor systems.

A similar organometallic synthetic methodology was used by A. Harriman et al. for the preparation of large molecular systems for light harvesting [99]. Other examples are molecules **61** and **62**, which contain donors and acceptors that were used for light harvesting (Figure 5) [23,100,101]. R. Ziessel et al. prepared also B-alkyne substituted oligomers [102,103]. Furthermore, M. J. Ortiz et al. used organometallic reagents to prepare a series of 4-substituted derivatives as potential laser dyes [104], whereas R. Ziessel et al. synthesized water-soluble red-emitting 4-substituted BODIPY derivatives for the sensing of bovine serum albumin [105].

**Scheme 20:**
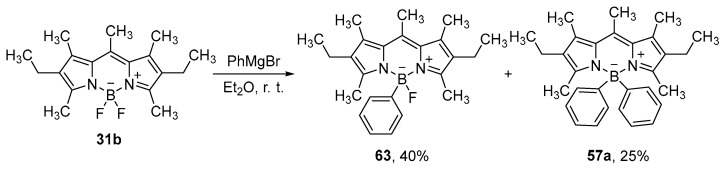
The substitution of fluorines in **31b** with a Grignard reagent.

4-Monosubstituted BODIPY compounds were prepared with less reactive Grignard reagents [96]. The reaction of compound **31b** with phenylmagnesium bromide at 0 °C produces monosubstituted compound **63** in a 40% yield (Scheme 20). At higher temperatures and with larger amounts of the Grignard reagents, the yields on monosubstituted product decrease, while the disubstituted product **57a** is formed.

## 4. Photochemical Reactivity of BODIPY Compounds on Boron

**Scheme 21:**
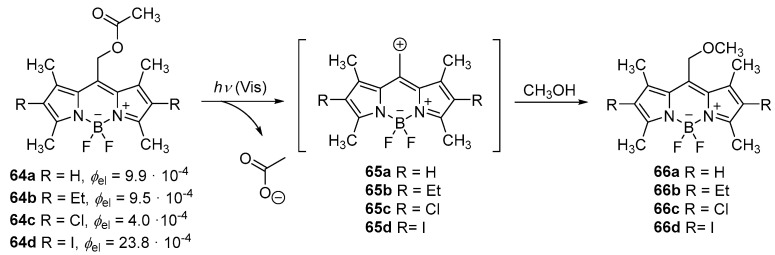
Photo-cleavage of acetate at the *meso*-methyl position for a series of BODIPY compounds and the corresponding quantum yields for the photo-elimination [115].

The number of described BODIPY compounds to date is very large, and most of them are characterized by exceptional photochemical stability. Therefore, the number of reports on photochemically reactive BODIPY compounds is limited, which are mostly connected to the development of photo-cleavable protective groups, known as photocages [106,107,108]. Photocages are used in biological research because they enable the manipulation of the activity of covalently bound substrates of interest and their temporally and spatially controlled release. While the appropriate functional group of the biologically active substrate is bound to the photocage, its biological activity is disabled, and it is activated by the release of the substrate after photoexcitation [109]. A. H. Winter et al., and P. Klan et al. synthesized a number of BODIPY compounds **64,** which are photo-cleavable at the *meso*-methyl group (Scheme 21) [51,110,111,112,113]. The advantage of BODIPY photocages compared to most organic chromophores is that the release of the substrate can be triggered by visible light, which is not harmful to healthy tissues and has a greater ability to penetrate through the tissue. They devoted a significant endeavor to elucidate the reaction mechanism, and it was proposed that the reaction mostly takes place via the triplet excited states and proceeds via the BODIPY carbocation, which is in its triplet ground state. The efficiency for the elimination depends on the groups on BODIPY, which facilitate the intersystem crossing and stabilize the carbocation and the leaving groups [51,110,111,112,113,114].

**Scheme 22:**
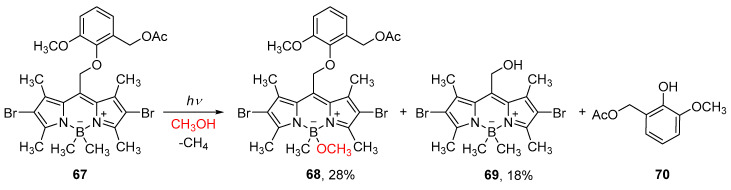
Competing photocleavage on boron and at the *meso*-position in BODIPY **67**.

When the BODIPY photocage has a poor leaving group at the *meso*-methyl position, such as phenoxyl, the cleavage at the boron competes with the elimination of the group from the *meso*-position. Thus, upon irradiation of **67**, two competing photoreactions take place (Scheme 22) [116].

**Scheme 23:**
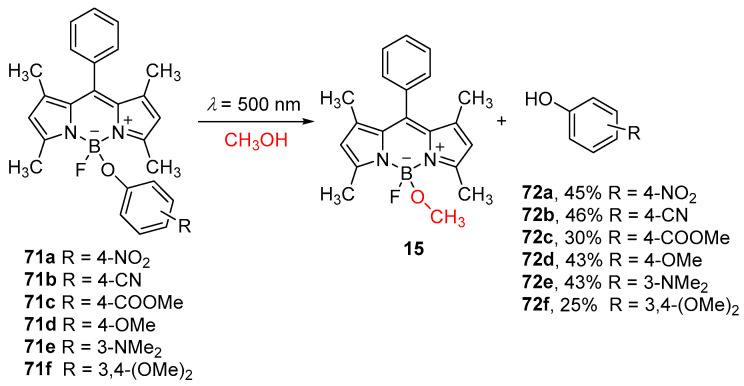
Photoreaction of BODIPY compounds **71** with phenolic substituent on boron.

The photocleavage on the boron atom was also reported by Y. Urano et al. Upon excitation by visible light BODIPY compound **71** with phenolic substituents on the boron atom underwent photoelimination reaction of the phenolic group (Scheme 23) [117]. It was assumed that photoexcitation gave rise to the radical cation of the phenolic groups and the radical anion of the BODIPY fluorophore, which were followed by the subsequent solvolysis of the B–O bonds [117].

The parameters affecting the elimination of phenolic groups from boron were additionally investigated. It was demonstrated that the efficiency of PET can be affected by the substitution of the BODIPY at the 2 and 6 positions or by substitution of the *para*-position of the phenol [118]. PET is more efficient if there are electronegative substituents at the positions 2 and 6 of the BODIPY and if phenols bear electron-donating substituents in the *para*-position. Furthermore, the efficiency of the elimination depends on the polarity of the solvent and is higher in non-polar solvents.

Photochemically reactive BODIPY compounds on boron were also used as self-immolative molecules, which was first described by J. A. Katzenellenbogen and co-workers [119]. Such systems consist of reactive carriers, scavenging links and substrates. The carriers can react with acids and bases, enzymes, or they can be excited by light and then release the linker and substrate. The reasons for the irreversible self-destruction of the linker are the increase in entropy of the system and the formation of thermodynamically stable products [120,121]. The most commonly used linkers are structures containing an aromatic ring with an electron-donating group (hydroxy-, amino- or thiol) which is in the *ortho*- or *para*-conjugation with a leaving group attached to the benzylic position of the ring. The presence of the electron-donating groups is necessary in order to reduce the energy barrier of dearomatization, and to form reactive intermediates: quinone methides, azaquinone methides, and tiaquinone methides. The destruction of the linker leads to the final release of the substrate and enables its biological actions [120,121].

**Scheme 24:**
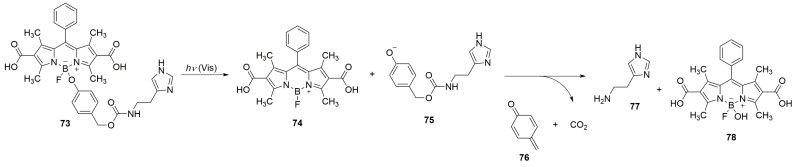
Photoreaction of BODIPY **73** on boron used for the release of histamine **77** in living cells.

An example of a self-immolative system, which is based on the photoreactivity of BODIPY on boron is molecule **73** (Scheme 24). The fluorophore is a reactive carrier to which the biologically active substrate histamine is bound by a *para*-hydroxybenzyloxycarbonyl self-destructing linker. The irradiation of **73** with visible light in living cells results in the release of phenolate **75**, which then decomposes into histamine **77**, CO_2_ and quinone methide **76** (Scheme 24) [117].

**Scheme 25:**
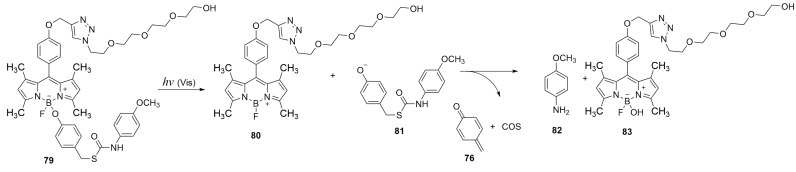
Photoreaction of BODIPY **79** on boron, applied in living nerve cells for the controlled release of H_2_S.

A similar approach for the photo-release in living cells is based on the photoreaction on boron of BODIPY compound **79**. It was used for the controlled release of COS, which is then decomposed to H_2_S (Scheme 25) [122].

## 5. Spectral and Photophysical Properties of 4-Substituted BODIPY Compounds

Several systematic investigations of spectral and photophysical properties of BODIPY compounds with substituents on the boron [114,118] have been reported, and some findings were mentioned in review articles [14,15,21,22,23,24,25,26,27,28,29,30,31]. Table 1 shows the spectral properties and photochemical reactivity of BODIPY compounds with different substituents of the boron. It is evident that the substituents do not significantly affect the spectral properties, but they can tune the photoreactivity at the *meso*-position, which is indicated from the differences in the reaction quantum yields [114].

The effect of the substituents on the boron on photophysical properties and on the photochemical reactivity were also studied by Y. Urano et al. Table 2 shows photophysical and photochemical data, which demonstrates that the 4-substituents do not affect significantly absorption maxima, but change the fluorescence quantum yield (*Φ*_F_) by almost two orders of magnitude [118]. The authors correlated the decrease in the fluorescence quantum yields with the increase in the energy of the HOMO orbitals of the phenolic groups, which they explained by PET from the phenolic groups to the BODIPY chromophore. It was suggested that the PET gives rise to the cleavage of the substituent on the boron, but the reported reaction quantum yields do not correlate with the quantum yields of fluorescence.

**Scheme 26:**
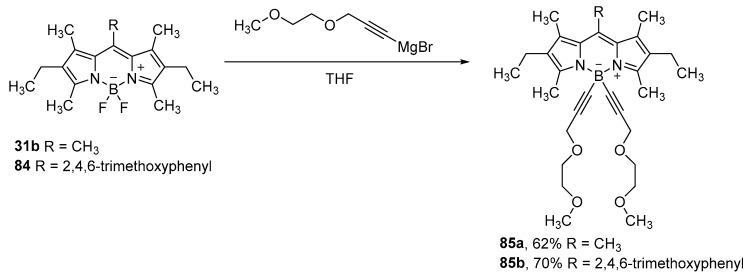
Synthesis of BODIPY dyes **85a** and **85b** for which the lasing properties were investigated.

The substitution of fluorines in BODIPY compounds changes the photophysical pathways by switching PET or energy transfer processes, and therefore, has been used in the design of organic photovoltaics [27]. Furthermore, the substituents on the boron also change the lasing properties of the dyes [123]. Thus, A. K. Ray et al. investigated photophysical properties and lasing ability of dyes **85a** and **85b** (Scheme 26 and Table 3). It is interesting that the introduction of the alkynyl substituents did not change the spectral and photophysical properties, but the lasing properties were improved. The improvement was connected with lower quantum yield of singlet oxygen formation for the 4-substituted BODIPY dyes, even though dye **85a** (*E*_ox_ = 0.95 V) was more easily oxidized than **31b** (*E*_ox_ = 1.02 V) by 70 mV [123].

**Figure 6:**
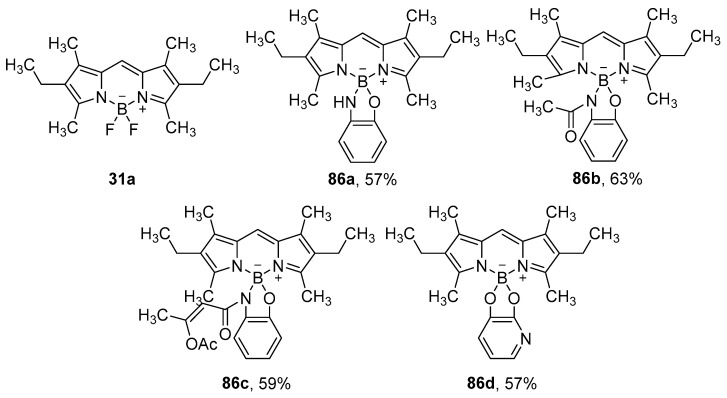
*O*,*B*,*O*- and *N*,*B*,*O*-substituted BODIPY compounds **86** showing delayed fluorescence.

J. G. Knight et al. investigated the photophysical properties of *O*,*B*,*O*- and *N*,*B*,*O*-substituted BODIPY compounds **86** shown in Figure 6 [124]. Contrary to the examples by Ray et al., they found that the fluorescence of the 4-substituted **86** was significantly quenched compared to the non-substituted **31a** (Table 4). Due to weak fluorescence of **87** in rigid matrix at 80 K, the fluorescence quenching was not associated with CT. Based on computations and temperature dependent fluorescence measurements, the authors proposed a reversible population of a dark trap state that is reversibly populated from the singlet, which leads to radiationless deactivation and delayed fluorescence [124].

Most BODIPY compounds are poorly soluble in polar solvents, especially water. Because of the planarity of the molecules, they aggregate due to *π*-*π* stacking [125]. By introducing hydrophilic, electronically charged or sterically large groups on the boron atom, which has a tetrahedral geometry, it is possible to effectively increase solubility and reduce aggregation [24,25,26,27,28,29,30,31,126,127]. Consequently, changing substituents on the boron in BODIPY compounds opens new avenues to finely tune physical properties, photophysical properties and reactivity, without affecting the spectral properties.

## 6. Summary and Perspective

This mini-review article highlights reactions on the boron atom in BODIPY compounds, which hitherto have not received significant attention. However, structural modification on the boron atom allows for the tuning of photophysical properties and different applications of new chromophoric systems. The reactions on the boron can be conducted under basic conditions, with strong nucleophiles such as alkoxides or organometallic reagents. Furthermore, the use of Lewis acids, which complex with the BF_2_ moiety, allows for different substitution protocols of the fluorines by nucleophiles. The use of BCl_3_ opens further avenues for the transformations, which include decomplexation and the formation of the free dipyrrins, the formation of the BCl_2_ complexes, and subsequent substitutions with N- C- or O-nucleophiles. The photochemical reactivity on boron is also possible and the photo-solvolysis reactions open opportunities for the development of new photocages and tools for photo-pharmacology. Consequently, the modifications of the BODIPY position 4 have yet to flourish, opening new horizons for further development of BODIPY chromophore in different scientific disciplines.

## Figures and Tables

**Table 1:** Spectral and photophysical properties of BODIPY compounds with different substituent of the boron [114].

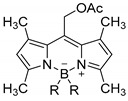					
R	λ_ab_/nm ^a^	λ_em_/nm ^b^	ε (×10^4^ M^−1^ cm^−1^) ^c^	Φ_r_ (%) ^d^	εΦ_r_/M^−1^ cm^−1^
phenol	522	540	9.2	0.31	285
BnOH	522	550	7.2	0.08	58
phenylacetic acid	523	547	10	0.08	80
catechol	524	538	5.9	0.12	71
benzotriazole	526	556	5.5	0.10	55
CN	521	536	4.6	0.08	37
Me	519	556	6.1	6.30	3842
OMe	522	540	4.0	0.20	80
F	517	529	7.1	0.14	99
CH_2_TMS	518	546	7.6	5.74	4362

^a^ Maximum in the absorption spectrum in CH_2_Cl_2_. ^b^ Maximum in the emission spectrum in CH_2_Cl_2_. ^c^ Molar absorption coefficient. ^d^ Quantum yield of the photoreaction determined by NMR in CDCl_3_-CD_3_OD (1:1) solution.

**Table 2:** Spectral and photophysical properties in CH_3_OH of BODIPY compounds with different phenoxyl substituents on the boron [118].

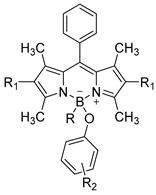				
R_1_	R_2_	λ_ab_/nm ^a^	Φ_F_ ^b^	Φ_r_ (×10^−4^) ^c^
Et	COOMe	523	0.62	ND
	CH_2_COOMe	523	0.42	19.8 ± 0.4
	Me	523	0.15	51.7 ± 2.1
	OMe	522	0.013	19.2 ± 0.4
H	COOMe	499	0.52	2.8 ± 0.2
	CH_2_COOMe	498	0.035	44.6 ± 3.6
	Me	498	0.029	53.8 ± 2.2
	OMe	500	0.011	25.6 ± 0.1
Cl	COOMe	524	0.32	6.5 ± 0.2
	CH_2_COOMe	522	0.038	19.3 ± 0.1
	Me	524	0.060	23.0 ± 3.3
	OMe	525	0.004	7.6 ± 1.6

^a^ Maximum in the absorption spectrum. ^b^ Quantum yield of fluorescence. ^c^ Quantum yield of the photoreaction. Data are presented as mean ± SEM from three independent experiments. ND = not determined due to the low uncaging efficiency.

**Table 3:** Photophysical properties of **31b**, **85a** and **85b** in ethanol [123].

Comp.	*λ*_ab_ /nm ^a^	*λ*_em_/nm ^b^	*ε* (×10^4^ dm^3^ mol^−1^ cm^−1^) ^c^	*ν*/cm^−1 d^	*Φ*_F_ ^e^	*τ*/ns ^f^
**31b**	517	534	8.3	616.0	0.84	6.43
**85a**	513	529	8.4	589.6	0.84	6.22
**86b**	523	533	9.4	358.7	0.81	6.61

^a^ Maximum in the absorption spectrum. ^b^ Maximum in the emission spectrum. ^c^ Molar absorption coefficient. ^d^ The Stokes shift. ^e^ Quantum yield of fluorescence. ^f^ Single excited-state lifetimes.

**Table 4:** Photophysical properties of **86** in methyltetrahydrofuran at rt [124].

Comp.	*λ*_ab_ /nm ^a^	*ε* (×10^4^ dm^3^ mol^−1^ cm^−1^) ^b^	*λ*_em_ /nm ^c^	*Φ*_F_ (%) ^d^	*τ*/ns ^e^
**31a**	495	9.347	508	0.94	5.6
**86a**	533	3.650	540	0.003	<0.03; 0.39
**86b**	533	2.832	543	0.001	0.04; 0.75
**86c**	533	2.766	549	0.004	0.04; 0.65
**86d**	535	2.816	540	0.015	0.18; 2.1

^a^ Maximum in the absorption spectrum. ^b^ Molar absorption coefficient. ^c^ Maximum in the emission spectrum. ^d^ Quantum yield of fluorescence. ^e^ Single and doubly excited-state lifetimes.
